# A Short Study Exploring the Effect of the Glycaemic Index of the Diet on Energy intake and Salivary Steroid Hormones

**DOI:** 10.3390/nu11020260

**Published:** 2019-01-24

**Authors:** Emad A.S. Al-Dujaili, Sophie Ashmore, Catherine Tsang

**Affiliations:** 1 Cardiovascular Science, Queen’s Medical Research Institute, Edinburgh University, Edinburgh EH16 4TJ, UK; 2 Dietetics, Nutrition & Biological Sciences, Queen Margret University, Edinburgh EH21 6UU, UK; sashmore@qmu.ac.uk; 3 Faculty of Health and Social Care, Edge Hill University, St Helens Road, Ormskirk, Lancashire L39 4QP, UK; catherine.tsang@edgehill.ac.uk

**Keywords:** diet, glycaemic index, testosterone, cortisol, steroid hormones

## Abstract

*Background:* The glycaemic index or load (GI or GL) is a concept for ranking carbohydrate-rich foods based on the postprandial blood glucose response compared with a reference food (glucose). Due to the limited research investigating the effect of the GI or GL of the diet on salivary steroidal hormones, this explorative short study was conducted. *Methods:* 12 female participants consumed a low GI and a high GI diet for three days each, followed by a washout period between each intervention. Saliva was collected at baseline, and following the low or high GI diets. Cortisol and testosterone concentrations were measured by enzyme-linked immuno-sorbent assay (ELISA). *Results:* GI and GL were significantly different between the low and high GI diets (*p* < 0.001). There was a small but significant increase in salivary cortisol after the high GI diet (7.38 to 10.93 ng/mL, *p* = 0.036). No effect was observed after the low GI diet. Higher levels of testosterone were produced after the low GI diet (83.7 to 125.9 pg/mL, *p* = 0.002), and no effect was found after the high GI diet. The total intake of calories consumed on the low GI diet was significantly lower compared to the high GI diet (*p* = 0.019). *Conclusions:* A low GI diet was associated with a small but significant increase in salivary testosterone, while a high GI diet increased cortisol levels. Altering the GI of the diet may influence overall energy intake and the health and wellbeing of female volunteers.

## 1. Introduction

There are numerous dietary regimes available to the general public, which are predominantly aimed at weight loss. One in five adults are classed as obese (body mass index (BMI) ≥ 30 kg/m^2^) in the UK which costs the National Health Service billions of pounds. Following the fall in popularity of high-protein, low-carbohydrate regimes, the glycaemic index (GI) diet is considered a diet of choice with the public. The GI diet was initially coined by Jenkins et al. [1] as a way of prescribing a diet for people with diabetes. Since then it has been developed as a dietary regime for weight loss [2,3,4]. Despite this, it is not currently endorsed by health authorities such as the British and American Dietetic Associations [5,6]. 

The GI is a concept for ranking carbohydrate-rich foods based on their postprandial blood glucose response compared with a reference food item, usually glucose or white bread. Per gram of carbohydrate, foods with a high GI produce a higher peak in postprandial blood glucose and a greater overall blood glucose response during the first 2 h after consumption compared to foods with a low GI [7]. A food is said to be of low GI if it has a GI of less than 55, medium GI if it has a GI of 56–69, and a high GI if it is 70 or greater [8,9]. The glycaemic load (GL) quantifies the overall glycaemic effect of a portion of food, rather than a 25g or 50g portion of carbohydrate, as with the GI. Much research of the GI of the diet has focused on improving blood glucose control in diabetes [10,11,12,13], weight reduction and obesity [4,14,15,16,17,18,19,20,21], female reproduction and polycystic ovary syndrome [22,23], blood lipids and cardiovascular health [5,17,24,25,26], and its role in cancer risk [27,28,29]. However, few studies have investigated its potential influence on steroidal hormones; cortisol and testosterone. Adrenal glucocorticoids (GC) are major modulators of multiple functions; including energy metabolism, stress responses, immunity, inflammation, cognitive function, mood, growth, reproduction and cardiovascular function [30]. Cortisol is the major naturally-circulating active GC secreted from the zona fasciculata, and its synthesis is primarily regulated by adrenocorticotropic hormone (ACTH) [31]. Cortisol exhibits a circadian rhythm; secretion is lowest in the evening and highest in the morning. Approximately 5–10% of circulating cortisol is free (biologically active), 75% is bound to corticosteroid-binding globulin (CBG), and 15% is bound to albumin. Cortisol has been extensively studied in relation to diabetes and insulin resistance [32], obesity [33], hypertension and cardiovascular disease (CVD) risk [34], immunosuppression [35], mood and wellbeing [36] and acute illness [37]. Testosterone, an androgen, is found in much lower concentrations in women compared to men. In men, it is the primary male sex hormone and an anabolic steroid produced by the testicular Leydig cells [38], however in women approximately two-thirds is the result of peripheral conversion of dehydroepiandrosterone (DHEA) and its sulphate to testosterone, with the remaining third being produced by the ovaries [39]. In both men and women, testosterone is involved in health and wellbeing, prevention of osteoporosis (men and postmenopausal women), and the ability to increase bone mineral density [40,41,42,43], increased libido [44] and increased muscle mass and strength [45]. Testosterone may act as a physiological antagonist of the catabolic stress hormone, cortisol. It is also thought to modulate mood and depression, the development of obesity [46] and breast cancer [47]. Normal serum concentrations of bioavailable testosterone in adult women are 66–791 pmole/L. Total serum testosterone in women range 0.347–1.873 nmole/L [48].

This study aims to investigate the consequences of changing the GI of the diet on salivary steroid hormones levels; cortisol and testosterone. 

## 2. Materials and Methods

### 2.1. Participants

Participants were recruited through an internal email moderator at Queen Margaret University, Edinburgh, United Kingdom. Twelve healthy females aged between 20–24 years volunteered to take part in the study. Eligibility criteria were: (a) female gender; (b) premenopausal and not taking contraceptive pill or hormone replacement therapy; (c) aged >20 years; (d) no acute illness in the previous month; and (e) not following a vegetarian, or any other medically prescribed diet. The study was approved by the research ethics committee at Queen Margaret University, Edinburgh, United Kingdom, code: 02020490/2012-GI/DNBS/QMU Ethical Committee. The study conformed to the guidelines set by the Declaration of Helsinki, and all participants provided written informed consent.

### 2.2. Study Design

The study followed a randomized controlled crossover design. All subjects followed a low GI and a high GI diet for three days each, separated by a washout period of three days. On days 1 and 2, participants completed diet diaries and also collected saliva samples on day 2 at 07.00hrs (before breakfast and before tooth brushing), at 09.00 hrs, 12.00 noon (before lunch) and 18.00 hrs (before dinner) to establish basal cortisol and testosterone values. On days 3–5, subjects consumed a low GI or high GI diet. Saliva was collected as above on day 5. On days 6–8, participants resumed their normal diet (washout period) to allow time for any changes in hormone concentrations to return to basal. On day 8, saliva was collected as above to determine if hormone concentrations had returned to pre-intervention levels. On days 9–11, participants crossed over to the subsequent GI diet fulfilling the crossover design, and saliva samples were collected at the aforementioned times on day 11. Participants were provided with information regarding the types of foods suitable for each GI diet, and were encouraged to make food choices consistent at each stage.

### 2.3. Saliva Samples

Steroid hormones, testosterone and cortisol, were measured in saliva due to the ease and non-invasive nature, and undue stress with this form of collection. It also reflects the unbound, active concentration of steroid hormones [49]. Studies have also shown good correlations between serum and saliva levels. With a salivary specimen one is able to collect multiple samples from the same individual at the optimum times for diagnostic information. This is of particular value for steroid hormones because they exhibit circadian or monthly variations. Blood concentrations of steroid hormones are several folds higher than saliva levels and, therefore, caution should be taken to avoid the problems of contamination from bleeding gums [49,50]. All saliva samples were collected by volunteers following written instructions on how to take the sample in the provided plastic tubes and store them in the fridge until their appointment when they were stored at −20 °C until processed.

### 2.4. Dietary Intervention

The diets were ad libitum leaving the choice for the volunteers to select the high GI or Low GI diet by themselves. A brief explanation of the GI was provided, but the complexities of the glycaemic load were not explained. For the purposes of this study a cut-off point defining low and high glycaemic index foods was not set, rather a list of foods were recommended to the subjects to give the greatest contrast, for example, potato crisps have a GI of 54 whereas corn chips have a GI of 72.

### 2.5. Assessment of Dietary Intake

Dietary intake was assessed using a three-day estimated food diary (basal: on 3 days prior to assessment). Food diaries were also completed during the high GI diet and the low GI diet using Win Diets Research software program (Version 2010, Robert Gordon University, Aberdeen, UK). Food portion sizes were determined using average or medium sizes (unless the subject specifically stated small or large) from published literature [51]. The GIs of food items were obtained from published tables [7], and were assigned to either low GI or high GI by matching to the most similar product; the reference food for all GIs was glucose. Where more than one GI was associated with a food item, for example, white bread has been studied 6 times providing GIs of 69–71, the GI of the product from the United Kingdom was used if possible, otherwise an average of all GIs for that product was used. If a brand name was specified in the food diary, the GI for this brand was used if available. The GI of a mixed dish where individual components were unclear was determined by its major source of carbohydrate, for example pasta with a creamy sauce was assigned the GI of plain pasta. The GL of each diet was also calculated based on the values presented by Foster-Powell et al. [7] according to the portion size of the food consumed [51]. The mean GI and GL of each diet was calculated and used for statistical analysis.

### 2.6. Laboratory Analyses

Saliva samples were processed, extracted and cortisol and testosterone were estimated by specific and sensitive enzyme-linked immuno-sorbent assays (ELISA), following the methods previously designed and published in our laboratory [50,52,53]. To reduce the effects of inter-assay variability, testosterone and cortisol saliva samples were assayed for each in duplicate in the same assay. The coefficients of intra- and inter-assay variation were 4.6–7.8% and 5.5–9.7% for testosterone ELISA respectively, and 3.8–7.2% and 4.8–10.4% for cortisol ELISA respectively. Minimum detection limit for testosterone and cortisol ELISAs were 2 pg/mL and 0.05 ng/mL, respectively.

### 2.7. Compliance

All participants completed the study and produced the full number of saliva samples requested. However, some volunteers provided samples up to 30 min outside the specified times. All saliva samples and diet diaries were included in statistical analysis.

### 2.8. Statistical Analysis

The mean values and standard deviations were calculated for testosterone and cortisol concentrations, and GI, GL and individual dietary components of each diet. Data was analysed using SPSS (Statistical Package for the Social Sciences, version 21, Chicago, IL, USA). To evaluate the variation between the three sets of data; basal, low GI and high GI, one way analysis of variance (ANOVA) was performed. Post hock comparisons using Bonferroni’s method was used to see which groups were statistically different. Paired-wise, 2-tail *t*-tests were also used to detect statistical significance in testosterone and cortisol concentrations, and changes in GI, GL and dietary components. *p* value of ≤0.05 was considered to be significant. Graphs were constructed in Microsoft Excel for windows XP version (Reading, UK).

## 3. Results

### 3.1. Participant Characteristics

Participant characteristics at baseline (Mean ± SD) are: Age was 21.1(0.9) years, BMI was 22.4(4.6) (kg/m^2^), alcohol intake was 3.2(2.4) units/week and exercise was 1.9(1.7) h/week. There were no significant differences between the above parameters at basal, low GI, washout or high GI diet.

### 3.2. Concentrations of Testosterone and Cortisol

**Figure 1:**
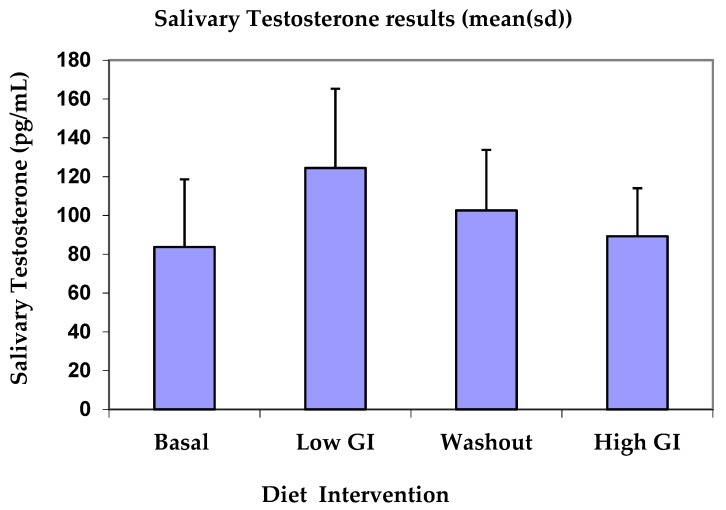
Testosterone concentration for basal, after low-GI diet, washout period and high-GI diet.

Table 1 shows basal, low GI, washout and high GI average salivary testosterone values per day. Testosterone concentrations in saliva increased significantly after the low GI diet (*p* = 0.002) compared to basal, and the difference between low GI and high GI testosterone concentration was significant (*p* = 0.009). There was no significant differences between basal and washout testosterone or basal and high GI testosterone concentrations. Figure 1 shows that testosterone concentrations increased from 83.71 pg/mL at basal to 125.86 pg/mL on the low GI diet, then declined to 100.57 pg/mL and 82.57 pg/mL for washout and high GI diet, respectively.

**Figure 2:**
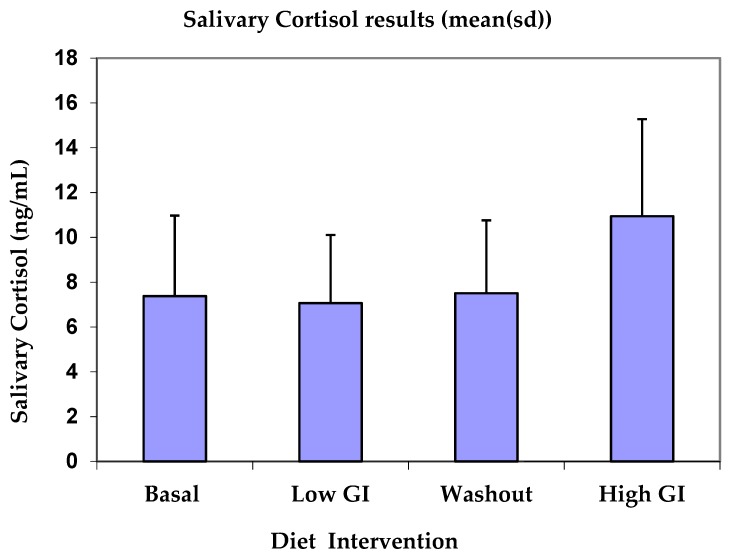
Cortisol concentration for basal, after low-GI diet, washout period and high-GI diet.

Table 2 shows basal, low GI, washout and high GI average salivary cortisol values per day. Salivary cortisol concentrations after high GI diet showed a significant increase compared to basal cortisol levels (*p* = 0.036). Differences between the low GI diet and basal or washout and basal were not significant. However, there was a significant difference between the low GI and high GI diet salivary cortisol (*p* = 0.012). Cortisol concentrations increased from 7.383 ng/mL at basal to 10.935 ng/mL on the high GI diet (Figure 2).

### 3.3. Individual Dietary Components

Table 3 shows the individual components of the diet of the basal, low-GI and high-GI diets. The GI of the low-GI diet was significantly lower compared to the high-GI diet (*p* < 0.001) and basal (*p* = 0.003), and the GI of the high-GI diet was significantly higher compared to basal (*p* = 0.022). The GL of the low-GI diet was significantly lower compared to the high-GI diet (*p* < 0.001) and basal (*p* = 0.001), and the GL of the high-GI diet was significantly higher compared to basal (*p* = 0.021).

Energy intake was significantly reduced on the low-GI diet compared to the high-GI diet (*p* = 0.022). There were no significant differences between the amount or percentage of energy supplied from fat, nor the amount of protein consumed. However a significantly higher proportion of energy came from protein on the low-GI diet (*p* = 0.01) compared to the high-GI diet. Significantly less carbohydrate was consumed on the low-GI diet compared to basal-GI diet (*p* = 0.002), and a significantly higher proportion of energy came from carbohydrate in the high-GI diet compared to low GI (*p* = 0.004). There was a significantly higher intake of starch and sugar on the high-GI diet compared to the low-GI diet (*p* = 0.004 and *p* = 0.012, respectively). There was no difference between the amount of sugar consumed at basal and on the high-GI diet. There was no significant differences in fibre content between the low- and high-GI diets; however there was a significantly lower fibre intake in the low-GI diet and high-GI diet compared to basal (*p* = 0.015 and *p* = 0.018, respectively).

## 4. Discussion

### 4.1. Dietary Recommendations

The low- and high-GI diet provided, respectively, 33% and 31% energy from fat, 17% and 14% energy from protein and 49% and 54% energy from carbohydrate. Both the low- and high-GI diets complied with the recommendations set by Scientific Advisory Committee on Nutrition—GOV.UK (SACN) Dietary Reference Values for Energy and carbohydrate (2011, 2015). A significantly lower energy intake was seen on the low-GI diet compared to the basal and high-GI diet. No advice was given to subjects about decreasing their energy intake and appeared to occur as a consequence of the low-GI diet, suggesting its potential for use as a weight control diet. The decreased energy intake could be due to the low-GI diet which is bulkier and more satiating than the basal diet, or the slower release of glucose into the blood stream with low-GI foods, resulting in smaller peaks in insulin release making the person to feel fuller for longer periods. Ajala et al. [12] reported that reducing blood glucose concentrations, induced weight loss, improved the lipid profile, and that low-GI and Mediterranean diets reduced cardiovascular risk in people with type 2 diabetes as evidenced by greater improvement in glycaemic control (glycated hemoglobin reductions). Schwingshackl and Hoffmann [16] provided evidence for beneficial effects of long-term interventions with a low-GI/GL diet in respect to fasting insulin and pro-inflammatory markers. Absolute fat intake did not change between diets, however a slightly higher percentage of energy came from fat on the low-GI diet and if the low-GI diet was to be used as a weight loss diet, the types of fat consumed have to carefully chosen. However, there is now evidence that people should avoid trans-fats which is more susceptible for oxidation. Subjects on low-GI diet days consumed less carbohydrate and more protein, and thus lower percentage energy from carbohydrate. There was no significant difference in carbohydrate intake or percentage energy between the basal- and high-GI diet, which indicates that the subjects basal diet is high in carbohydrate.

The GI of the two diets was significantly different. The low-GI diet had an average GI of 42 (well within the low-GI category of <55), which was raised to 59 on the high-GI diet, however this is at the lower end of the medium GI category (GI = 56–69). This small, but significant, rise in GI is probably due to the large amounts of fruits and vegetables consumed on the high-GI diet, thus lowering the average, despite the conscious efforts of the subjects to choose a high-GI diet. Subjects were not advised to limit their intake of fruits and vegetables as this has implications on health, however in hindsight this should have been recommended. Although subjects were not given information regarding the GL, this was significantly decreased on the low-GI diet compared to the high-GI diet, indicating that the portion sizes of carbohydrate-rich foods decreased, or that carbohydrate containing foods were simply cut down. We demonstrated that there was no significant difference in the fibre intake between the low-GI and high-GI diets, however increased dietary fiber intake was associated with better glycemic control and improved CVD risk factors including chronic kidney disease suffering from type 2 diabetes [54].

### 4.2. Testosterone

Because of the often extremely low levels of testosterone in female saliva, one of the particular requirements for its quantitative determination is that the assay be especially sensitive. The in house testosterone ELISA reagents and conditions were optimised to produce the required sensitivity of 2 pg/mL [50]. Testosterone concentrations were significantly increased by the low-GI diet compared to basal and high GI diet, and this might be beneficial, in particular, to overweight and obese people. Santos et al. [55] compared the effects of a protein and an energy restricted diets on serum testosterone levels in rats, and found that only the energy-restricted group showed a significant decrease in serum testosterone concentrations. Although the low-GI diet provided fewer calories, a decrease in testosterone concentrations was not seen. However, it cannot be assumed that the energy deficit of 400 kcal on the low GI diet did not affect the results. It was reported that low-fat diets cause a reduction in total and free testosterone [47,56], but in our study, there was no significant difference between fat intake, percentage energy derived from fat and the GI of the diet. Some researchers reported an association between low testosterone and obesity in men [57], and that testosterone was found to increase adiposity, leading to a cycle of metabolic complications [58]. Moreover, sugar-sweetened beverage intake was associated with low serum testosterone in men [59], and a high-GI diet was found to induce a reduction in total and free testosterone levels in men [60].

### 4.3. Cortisol

The actions of cortisol on carbohydrate, protein and fat metabolism are well known, however the effects of these macronutrients on cortisol production is less well understood. Stimson et al. [61] found no differences in plasma cortisol between high carbohydrate meals with a high GI or low GI and changes in cortisol concentrations usually occur slowly over a period of few days. However, our study showed an increase in cortisol levels after the high-GI diet compared to the basal- or low-GI diet. There are many variables that influence cortisol production, including stress, diet and feeding times, sleep pattern and light-dark exposure, however cortisol levels are mostly influenced by stress, whether it is physical, such as illness, or psychological, such as anxiety [31,62].

Venkatraman et al. [63] reported that plasma cortisol levels were elevated on a 40% fat diet and post endurance exercise. Repeated elevation of cortisol can lead to weight gain via visceral fat storage [64,65], and reducing the glycaemic impact (GI and GL) of the diet was found to restore hormone balance and maintenance of a healthy weight [66], particularly, for women with polycystic ovary syndrome [23]. High visceral fat mass is now widely accepted to be a risk factor for the development of cardiometabolic diseases, and low testosterone levels could also be important [67]. In addition, 11β-HSD1 (the enzyme responsible for activating cortisone to cortisol) was found to be selectively elevated in adipose tissue where it contributes to metabolic complications [68].

### 4.4. Limitations of Study

There were several limitations of the present study. Food combinations most likely would have affected the GI; a lower GI food eaten with a higher GI food lowers the overall GI, and high fat foods usually have a lower GI than expected. Another limitation was the great inter-subject variation in the GI of foods and day-to-day variation of the glycemic response in subjects. Due to the exploratory nature this was a short-term study involving a small number of participants, with the dietary intervention periods limited to 3 days, which was perhaps not long enough to see the full extent of their effects on hormone concentrations. Moreover, accurately recording food intake is inherently difficult as perceptions of portions differ between individuals, and people tend to lie about what they have eaten or modify their eating habits when having to record food intake. Participants noted that it was difficult to adhere to the low-GI diet as many products habitually eaten have a high GI. This observation was confirmed by the fact that the GI of the basal diet was closer to the GI of the high-GI diet than the low-GI diet. Comprehensive lists of GI values may have aided compliance yet subjects also found it difficult to estimate the GI of some products that were eaten frequently in hidden foods e.g., sauces.

### 4.5. Conclusions

The take-home message from this exploratory short-term and small study is that lowering the GI and GL of the diet may produce better health in lowering the energy intake, and in particular, sugar intake. Modulating the levels of testosterone and the stress hormone, cortisol, might be of benefit to mood and well being. Longer-term studies are needed to enable the observed changes to be detected on a large scale and thus recommendation to check the GI of the diet would then be justified. However, if the GI of diet is to be followed by many people in this country, better labelling and more GI testing of foods are required which may prove to be costly and time consuming.

## Figures and Tables

**Table 1:** Testosterone values at basal, low glycaemic index (GI), washout and high-GI diet.

Average Testosterone/day	Mean (pg/mL)	Standard Deviation	*p* Value
Basal Low GI	83.71 125.86	34.93 39.71	0.002
Basal Washout period	83.71 100.57	34.93 28.32	0.339
Basal High GI	83.71 82.57	34.93 20.8	0.896
Low GI High GI	125.86 82.57	39.71 20.8	0.009

**Table 2:** Cortisol values of basal, low GI, washout and high-GI cortisol.

Average Cortisol/day	Mean (ng/mL)	Standard Deviation	*p* Value
Basal Low GI	7.383 7.065	3.588 3.036	0.733
Basal Washout period	7.383 7.518	3.588 3.248	0.876
Basal High GI	7.383 10.935	3.588 4.337	0.036
Low GI High GI	7.065 10.935	3.036 4.337	0.012

**Table 3:** Dietary components on basal, low-GI and high-GI diets.

Dietary Component	Variables Compared	Mean	Standard Deviation	*p* Value
Energy intake (kcal/day)	Basal energy intake low-GI energy intake	1925 1509	297.88 258.14	0.019
	Basal energy intake high-GI energy intake	1925 1841	297.88 222.11	0.492
	Low-GI energy intake high-GI energy intake	1509 1841	258.15 222.11	0.022
Fat intake (g/day)	Basal fat intake low-GI fat intake	63.6 55.9	23.48 13.29	0.432
	Basal fat intake high-GI fat intake	63.6 63.9	23.48 10.11	0.974
	Low-GI fat intake high-GI fat intake	55.9 63.9	13.29 10.11	0.172
% energy from fat	Basal percent energy low-GI percent energy from fat	30 33	7.15 3.02	0.258
	Basal percent energy high-GI percent energy from fat	30 31	7.15 2.86	0.607
	Low-GI percent energy high-GI percent energy from fat	33 31	3.02 2.86	0.148
Protein intake (g/day)	Basal protein intake low-GI protein intake	58.5 64.1	15.85 9.09	0.467
	Basal protein intake high-GI protein intake	58.5 63.5	15.85 7.51	0.564
	Low-GI protein intake high-GI protein intake	64.1 63.5	9.09 7.51	0.869
Percent energy from protein	Basal percent energy low-GI percent energy from protein	13 17	1.50 2.12	0.005
	Basal percent energy high-GI percent energy from protein	13 14	1.58 2.58	0.275
	Low-GI percent energy high-GI percent energy from protein	17 14	2.18 2.58	0.010
Carbohydrate intake (g/day)	Basal carbohydrate intake low-GI carbohydrate intake	278.8 197.8	40.00 33.46	0.002
	Basal carbohydrate intake high-GI carbohydrate intake	278.8 267.2	40.04 43.55	0.322
	Low-GI carbohydrate intake high-GI carbohydrate intake	197.8 267.2	33.47 43.55	0.004
Percentage energy from carbohydrate	Basal % energy low-GI % energy from carbohydrate	56 49	8.83 3.33	0.026
	Basal % energy high-GI % energy from carbohydrate	56 54	8.83 4.07	0.420
	Low-GI % energy high-GI % energy from carbohydrate	49 54	3.33 4.07	0.015
Starch intake (g/day)	Basal starch intake low-GI starch intake	157.6 117.9	28.53 16.47	0.008
	Basal starch intake high-GI starch intake	157.6 147.8	28.53 21.12	0.278
	Low-GI starch intake high-GI starch intake	117.9 147.8	16.47 21.12	0.004
Sugar intake (g/day)	Basal sugar intake low-GI sugar intake	113.8 78.9	21.03 21.45	0.006
	Basal sugar intake high-GI sugar intake	113.8 114.1	21.03 29.29	0.957
	Low-GI sugar intake high-GI sugar intake	78.9 114.1	21.45 29.29	0.012
Fibre intake (g/day)	Basal fibre intake low-GI fibre intake	12.9 10.3	3.14 1.55	0.015
	Basal fibre intake high-GI fibre intake	12.9 9.1	3.14 1.90	0.018
	Low-GI fibre intake high-GI fibre intake	10.3 9.1	1.55 1.90	0.206
Glycaemic Index (GI)	Basal GI low-GI GI	51 42	4.56 2.43	0.003
	Basal GI high-GI GI	51 59	4.56 3.07	0.022
	Low-GI GI high-GI GI	42 59	2.43 3.07	<0.001
Glycaemic load (GL)	Basal GL low-GI GL	133 72	18.65 14.78	0.001
	Basal GL high-GI GL	133 149	18.65 21.25	0.021
	Low-GI GL high-GI GL	72 149	14.78 21.25	<0.001

## Abbreviations

ACTH	adrenocorticotropic hormone
BMI	body mass index
CBG	corticosteroid-binding globulin
CVD	cardiovascular disease
DHEA	dehydroepiandrosterone sulphate
ELISA	enzyme linked immuno-sorbent assay
GC	glucocorticoids
GI	glycaemic index
GL	glycaemic load
SHBG	sex hormone binding globulin
SPSS	Statistical Package for the Social Sciences
